# Polyelectrolyte Microcapsules—A Promising Target Delivery System of Amiodarone with the Possibility of Prolonged Release

**DOI:** 10.3390/ijms24043348

**Published:** 2023-02-08

**Authors:** Aleksandr L. Kim, Egor V. Musin, Munojat J. Oripova, Yulia I. Oshchepkova, Shavkat I. Salikhov, Sergey A. Tikhonenko

**Affiliations:** 1 Institute of Theoretical and Experimental Biophysics Russian Academy of Science, Institutskaya St., 3, 142290 Puschino, Moscow Region, Russia; 2 Institute of Bioorganic Chemistry named after O.Sodikov Academy of Sciences of the Republic of Uzbekistan, M. Ulugbek Str., 83, Tashkent 100125, Uzbekistan

**Keywords:** polyelectrolyte microcapsules, amiodarone, encapsulation, drug delivery, target delivery

## Abstract

Atrial fibrillation is one of the most common cardiac arrhythmias. Pharmacological preparations are used for treatment to control heart rate and rhythm. Amiodarone is one of these highly effective preparations, but, at the same time, it has significant toxicity and nonspecific accumulation in tissues. The drug delivery system based on polyelectrolyte microcapsules is one of the solutions. For this purpose, we compared different encapsulation methods of amiodaron: monoammonium salt of glycyrrhizic acid (Am:MASGA) complex (molar ratio 1:8). The concentration of amiodarone was determined by spectrophotometric methods at 251 nm. It has been shown that the co-precipitation method allows capturing 8% of Am:MASGA by CaCO_3_ microspherulites, which is not sufficient for the long-acting drug. The adsorption method allows encapsulating more than 30% of Am:MASGA into CaCO_3_ microspherulites and polyelectrolyte microcapsules CaCO_3_(PAH/PSS)_3_, but, at the same time, an insignificant amount of substance is released into the incubation medium. The development of delivery and long-acting drug system based on such methods are not inexpedient. The most appropriate encapsulation method of Am:MASGA is the adsorption method into polyelectrolyte microcapsules with complex interpolyelectrolyte structure (PAH/PSS)_3_. Such a type of PMC adsorbed about 50% of the initial amount of the substance and 25–30% of Am:MASGA was released into the medium after 115 h of incubation. The adsorption of Am:MASGA by polyelectrolyte microcapsules has electrostatic nature as evidenced by the acceleration of the release by 1.8 times as ionic strength increases

## 1. Introduction

Atrial fibrillation (AF) is the most common cardiac arrhythmia associated with the interruption of the electrical conductivity of atriums [1,2,3]. This is a disease that has affected more than 33 million people all over the world, and every 10 years that number doubles according to American Heart Association Statistics Committee [4,5,6]. In addition to symptoms associated directly with AF, this disease leads to serious complications such as increasing embolic insult possibility, hemodynamic dysfunctions, and cardiomyopathy-mediated tachycardia [7,8,9]. As a result, such a disease significantly contributes to morbidity and mortality of the population, and it significantly increases expenses on the health care system (USA—over 33 billion dollars) [10,11].

One of the main principles of atrial fibrillation treatment is the use of pharmacological preparations to control heart rate and rhythm [12]. One of those drugs is Amiodarone [13]. Amiodarone is a lipophilic substance used to treat different supraventricular and ventricular arrhythmias, as well as for the treatment of AF [14], due to its effect on the electrophysiological processes in myocardia [15]. The distinctive features of the remedy are its high effectiveness and low rate of relapses compared with other drugs (e.g., Dronedarone, Sotalol) [16]. However, Amiodarone use is associated with the high toxicity of the remedy and its non-specific accumulation in tissues [17,18]. Patients continuously receiving amiodarone have a higher amount of the substance accumulated in non-target tissues (by 10–500 times) than in cardiac tissues [19,20]. The treatment has pronounced cardiotoxicity and pulmonary toxicity, which leads to the emergence of pulmonary fibrosis, hypo/hyperthyroidism, and liver dysfunction in 18% to 75% of cases [20,21,22]. In this way, new solutions are needed to improve the therapeutic effect of amiodarone while reducing side effects.

One of the solutions is the implantation of gel based on polyethylene glycol contained amiodarone into the atrial epicardium through the pericardial space in a minimally invasive surgery. It was shown that the non-target amount of remedy in the organs of the liver, lungs, thyroid gland, and adipose tissue was significantly lower in animals that received epicardial amiodarone gel compared with the animals that received an injection of a pure drug directly into the circulatory system [23,24]. The disadvantage of this solution is the invasive intervention during gel implantation, which may be not acceptable to all patients. Another effective delivery way of amiodarone is to use nanoparticles as a drug carrier. For example, Maaz S. Ahmed et al. [25] used nanoparticles made of succinyl-β-cyclodextrin crosslinked with L-lysine to bind amiodarone and its increase affinity for cardiac macrophages. Biodistribution analyzes showed the accumulation of nanoparticles in the heart due to rapid uptake by cardiac macrophages. It led to a 250% increase in the selectiveness of drug delivery to cardiac tissue compared to the pure drug. However, at the same time, such a system does not allow prolong of the drug’s action. Ying Zhuge et al. attempted to solve this problem by incorporating amiodarone into liposomes, as a result, the medicine has a gradual release and more selective accumulation in tissues. However, this selectivity was significantly lower than in the case of using nanoparticles [26].

Thus, the development of a delivery system for amiodarone with prolonged release and diminished side effects is relevant.

A possible solution to the problems described above is the development of a target delivery system based on the polyelectrolyte microcapsules [27,28]. Such microcapsules are obtained with layer-by-layer adsorption of oppositely charged polyelectrolytes on a micron-sized colloidal particle [29]. Due to the controlled variability of the composition of the PMC shell, optimal conditions can be selected for the incorporation of amiodarone and its gradual release in the target organ. In this connection, we studied the possibility of amiodarone encapsulation in different types of polyelectrolyte microcapsules with its gradual release into the medium.

## 2. Results and Discussions

**Figure 1:**
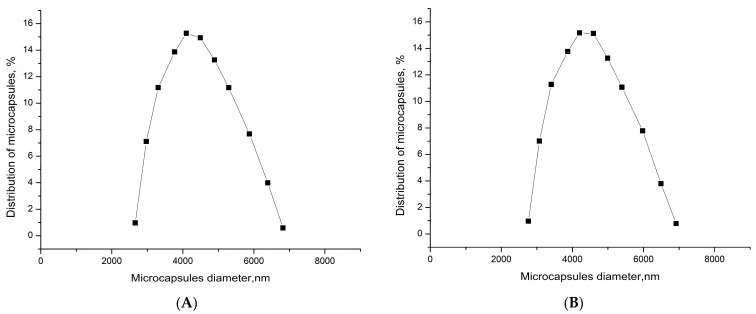
Size distribution of CaCO_3_ microspherulites formed in the co-precipitation (**A**) and adsorption (**B**) method.

In this work, the possibility of encapsulation of the amiodarone: monoammonium salt of glycyrrhizic acid (Am:MASGA) complex (molar ratio 1:8) in various types of micron-sized carriers was studied. One such carrier is polyelectrolyte microcapsules (PMC). The first step of the polyelectrolyte microcapsules creation is the formation of a core containing the target substance. The core of polyelectrolyte microcapsules is CaCO_3_ microspherulite with a complicated nanoporous structure. The size distribution is demonstrated in Figure 1.

**Figure 2:**
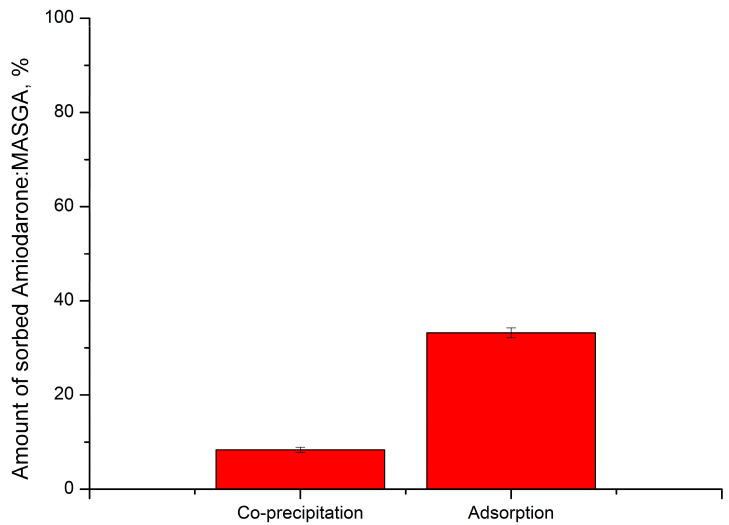
Amount of incorporated Am:MASGA by CaCO_3_ microspherulites with co-precipitation and adsorption methods.

The encapsulation of Am:MASGA is carried out either by the method of co-precipitation or adsorption [30,31]. In this regard, initially, the possibility of incorporating Am:MASGA into CaCO_3_ microspherulites by the method of co-precipitation and adsorption was studied. In the case of co-precipitation, calcium carbonate microspherulites were formed in the presence of Am:MASGA. Further, CaCO_3_ with Am:MASGA were co-precipitated, the supernatant was taken and the absorbance of Am:MASGA at 251 nm was determined spectrophotometrically. In the case of adsorption, sodium carbonate microspherulites were formed separately, after which they were incubated in an Am:MASGA solution for 1 h. After incubation, the supernatant was taken and the absorbance of Am:MASGA at 251 nm was determined spectrophotometrically. The results obtained are shown in Figure 2.

As can be seen from the figure, the co-precipitation method makes it possible to include Am:MASGA into CaCO_3_ microspherulites with no more than 8%. At the same time, the amount of the included substance by the adsorption method is much higher and accounts for more than 33%. Thus, it can be concluded that the adsorption method is a more appropriate method for incorporating Am:MASGA.

**Figure 3:**
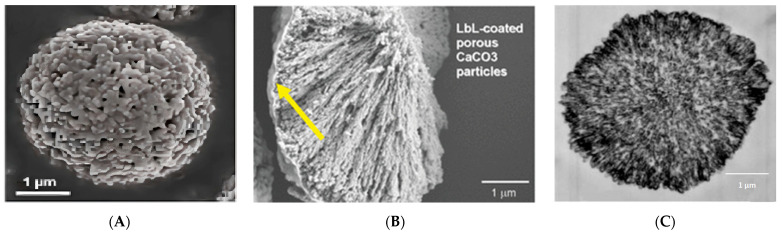
Photographs of electron microscopy of various types of carriers: (**A**)—photograph of scanning electron microscopy of CaCO_3_ microspherulites [33], (**B**)—scanning electron microscopy photograph of CaCO_3_ coated with a polyelectrolyte shell [31], (**C**)—electron microscopy photograph of an ultrathin section of a polyelectrolyte microcapsule [34].

At the next stage, the possibility of encapsulation of Am:MASGA into various types of carriers by the adsorption method was studied in more detail. For this, CaCO_3_ microspherulites (Figure 2A) and 2 types of polyelectrolyte microcapsules were used: PMC with a not removed CaCO_3_ core (Figure 2B)—CaCO_3_/(PAH/PSS)_3_ and PMC with a removed CaCO_3_ core and with the complex polyelectrolyte structure [32] (Figure 2C)—(PAH/PSS)_3_). These structures are shown in Figure 3.

### 2.1. Am:MASGA Incorporating into CaCO_3_ Microspherulites

**Figure 4:**
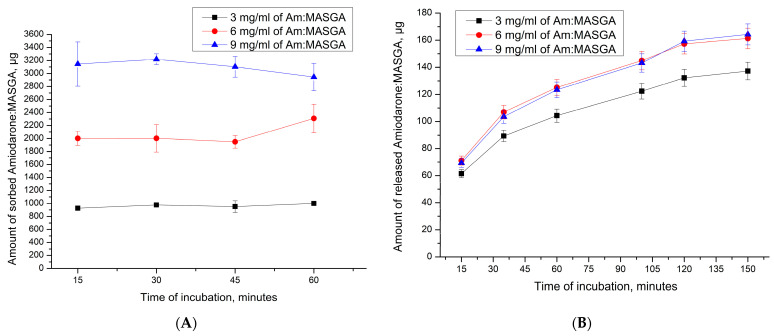
Dynamics of sorption and release of Am:MASGA by CaCO_3_ microspherulites. (**A**)—the amount of sorbed substance, μg; (**B**)—the amount of released substance, μg.

At first, the possibility of Am:MASGA incorporating into CaCO_3_ microspherulites was studied. For this, the sorption ability of Am:MASGA by CaCO_3_ microspherulites was determined. For this purpose, CaCO_3_ microspherulites were incubated in Am:MASGA with different concentrations for 1 h, and the amount of the sorbed substance was measured every 15 min. The results are presented in Figure 4A.

As can be seen from Figure 4A, the amount of sorbed substance does not change over time and reaches equilibrium in the first 15 min of incubation. In addition, an increase in the Am:MASGA concentration in the incubation solution lead to a linear and proportional increase of the substance mass sorbed by microspherulites (Figure 4A). In this case, regardless of the concentration of the solution, about 30–35% of the substance from the initial concentration is sorbed. The effect described above is presumably related to the physical nature of the absorption of Am:MASGA by spherulites [35]. This is also confirmed by the presence of a developed nanoporous structure inside CaCO_3_ microspherulites, which provides polar interaction between Am:MASGA and the crystal surface [36,37,38].

Subsequently, the dynamics of the release of Am:MASGA from these microspherulites were studied. For this purpose, CaCO_3_ microspherulites were incubated in a distilled water solution for 1 h, and the amount of the released substance was measured every 15 min. The results are presented in Figure 3B. As can be seen from Figure 4B, about 70 µg of Am:MASGA is released from CaCO_3_ microspherulites during the first minutes of incubation in water. In the future, the release of the substance doesn’t exceed 160 μg with a tendency towards the plateau. According to the results obtained above, it can be seen that the release of Am:MASGA from spherulites stops after only 2 h of incubation with the release of about 9% of the initially included substance. Such a low release efficiency makes it inexpedient to develop a delivery system and prolonged release system of Am:MASGA based on CaCO_3_.

**Figure 5:**
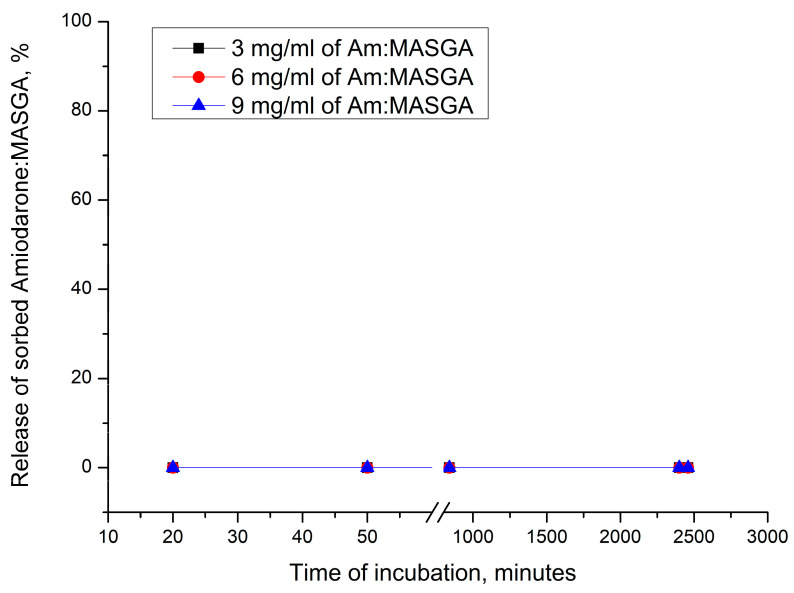
The release dynamics of Am:MASGA from polyelectrolyte microcapsules (PAH/PSS)_3_ with dissolved CaCO_3_, where 100% was taken as the amount of encapsulated substance.

In this regard, it was proposed to cover the microspherulites with a polyelectrolyte shell and remove the CaCO_3_ core, which may allow the slow release of the encapsulated substance. For this, Am:MASGA was incorporated into CaCO_3_ microspherulites by adsorption, covered with a polyelectrolyte shell (PAH/PSS)_3_, and the spherulite core was removed using EDTA. For this purpose, PMCs were incubated in a distilled water solution, and the amount of the released substance was measured every 15 min. The results are presented in Figure 5.

As can be seen from Figure 5, Am:MASGA is not released into the incubation solution, it is probably due to the loss of Am:MASGA during the dissolution of CaCO_3_ microspherulites with EDTA. The amount of substance released during incubation in EDTA solution cannot be determined, because the absorption spectrum of EDTA overlaps the spectrum of Am:MASGA. However, this type of encapsulation in PMC is not suitable for the purpose of our work, because ultimately Am:MASGA does not release into the incubation solution, and therefore it is not an urgent task to determine the amount of the lost substance during incubation in EDTA.

### 2.2. Encapsulation of Am:MASGA by Adsorption into CaCO_3_ Microspherulites Coated with a Polyelectrolyte Shell

**Figure 6:**
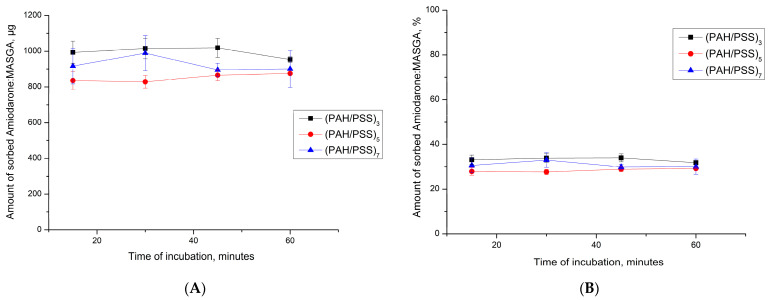
Dynamics of sorption of Am:MASGA (3 mg/mL) by CaCO_3_ microspherulites coated with a polyelectrolyte shell of the composition (PAH/PSS)n, where “n” is the number of pairs of layers equal to 3, 5 or 7. (**A**)—the amount of sorbed substance in μg, (**B**)—the amount of sorbed substance in%.

Subsequently, it was proposed to encapsulate Am:MASGA by adsorption into another type of microcapsules—CaCO_3_ microspherulites coated with a polyelectrolyte shell. Coating particles with a multilayer polyelectrolyte shell can increase the sorption of Am:MASGA and prolong its release into the solution. This is possible both due to the release of Am:MASGA from the polyelectrolyte shell and from the CaCO_3_ core. The first step to confirm this hypothesis is to study the dynamics of Am:MASGA CaCO_3_ adsorption by microspherulites coated with a polyelectrolyte shell of composition (PAH/PSS)_n_, where “n” is the number of pairs of layers equal to 3, 5, or 7. The results are presented in Figure 6.

As can be seen from Figure 6, CaCO_3_ microspherulites coated with a polyelectrolyte shell of the composition (PAH/PSS)_n_ sorbed about 900 ± 100 μg Am:MASGA, which is approximately 30% of the substance from the initial amount (3 mg/mL). In addition, it can be seen that an increase in the number of polyelectrolyte layers does not lead to an increase in the sorbed substance. This is presumably due to the high density of the polyelectrolyte shell and the low accessibility of amiodarone to the internal space of spherulites, where most of the polyelectrolyte is located.

**Figure 7:**
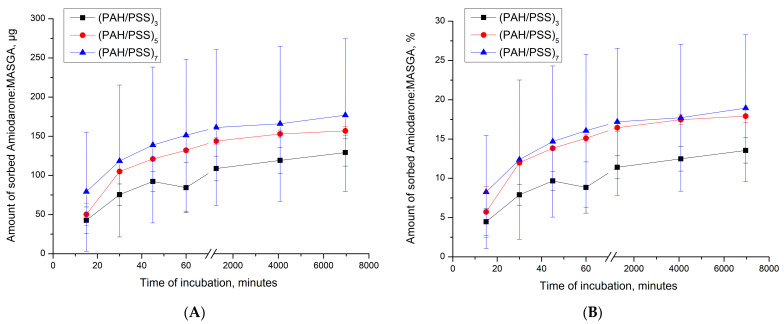
Am:MASGA release dynamics from CaCO_3_/(PAH/PSS)_n_ microcapsules, where n is the number of pairs of layers equal to 3, 5 or 7. (**A**) is the amount of released substance in μg, (**B**) is the amount of released substance in %, where 100% took the amount of substance included.

Subsequently, the dynamics of the release of Am:MASGA from CaCO_3_/(PAH/PSS)_n_ was studied to evaluate the release of the substance over time. For this purpose, PMC CaCO_3_/(PAH/PSS)_n_ were incubated in a distilled water solution, and the amount of the released substance was measured every 15 min. The results are presented in Figure 7.

As can be seen from the figure, about 10–15% Am:MASGA is slowly released from microcapsules during the 1st hour of incubation, regardless of the number of polyelectrolyte layers. After 116 h of incubation, a small amount of Am:MASGA is released; the release of the substance stops. Thus, PMCs of the CaCO_3_/(PAH/PSS)_n_ type are not suitable for the encapsulation of Am:MASGA, because the release of the substance is observed only within 1 h and does not exceed 15% of the encapsulated substance.

### 2.3. Encapsulation of Am:MASGA by Adsorption into Polyelectrolyte Microcapsules (PAH/PSS)_n_

**Figure 8:**
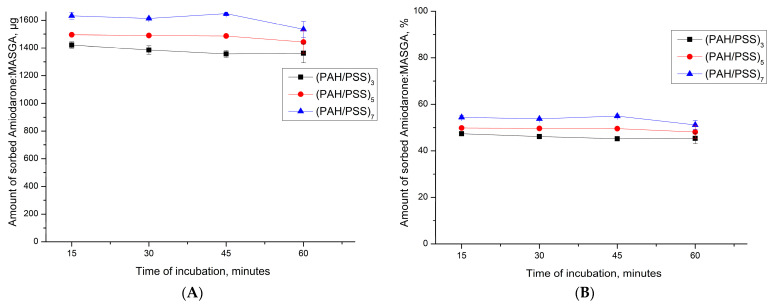
Am:MASGA sorption dynamics of PMC of the (PAH/PSS)_n_ structure, where n is the number of layer pairs equal to 3, 5, or 7. (**A**) is the amount of sorbed substance in µg, (**B**) is the amount of sorbed substance in %.

Subsequently, it was proposed to encapsulate Am:MASGA by adsorption into another type of capsule—(PAH/PSS)_3_ with a complex polyelectrolyte structure. Such microcapsules have a more developed nanoporous structure and a larger number of uncompensated polyelectrolyte complex regions than inside PMC CaCO_3_/(PAH/PSS)_n_. These properties will presumably increase the interaction of PMC with the incubation medium, which will increase the prolonged release of the substance and increase the sorption capacity of Am:MASGA. For this, the adsorption dynamics of Am:MASGA of the PMC structure (PAH/PSS)_n_ were studied, where n is the number of pairs of layers equal to 3, 5, or 7. The results are shown in Figure 8.

As can be seen from Figure 8, PMC of the (PAH/PSS)_n_ structure sorbed about 1500 ± 100 μg of Am:MASGA, which is approximately 50% of the substance from the initial amount. In addition, it can be seen that an increase in the number of polyelectrolyte layers does not lead to a significant increase in the sorbed substance. This effect is presumably due to the fact that the sorption of a substance greater depends on the polyelectrolyte located in the internal volume of PMC, which contains most of the uncompensated polyelectrolyte complex. While the shell, which is formed after the 10th layer [34,39,40] has a dense structure and polyelectrolytes are more compensated.

**Figure 9:**
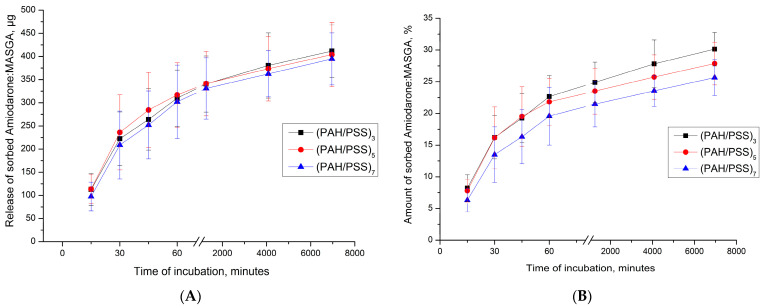
Am:MASGA release dynamics from microcapsules (PAH/PSS)_n_, where n is the number of pairs of layers equal to 3, 5 or 7. (**A**) is the amount of released substance in μg, (**B**) is the amount of released substance in %, where 100% took the amount of included substance.

Subsequently, the dynamics of the release of Am:MASGA from PMC of the (PAH/PSS)_n_ structure was studied. For that purpose, the microcapsules (PAH/PSS)_n_ were incubated in water, and the concentration of the released substance was measured every 15 min. The results obtained are shown in Figure 9.

As can be seen from the figure, after 115 h of incubation, about 25–30% Am:MASGA is slowly released from microcapsules, regardless of the number of polyelectrolyte layers. That is, this type of capsule is suitable for encapsulating Am:MASGA, since there is a slow release of this substance, which is excellent for prolonging the action of the drug. In this case, it is sufficient to use PMC of the composition (PAH/PSS)_3_, since the use of more layers is impractical.

**Figure 10:**
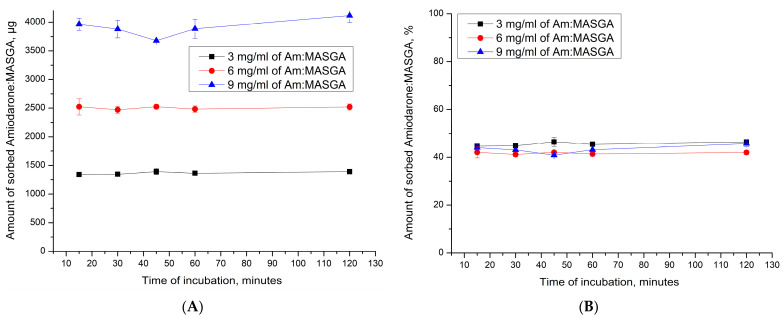
Dynamics of sorption of Am:MASGA with different concentrations by PMC (PAH/PSS)_3_. (**A**) is the amount of sorbed substance in μg; (**B**) is the amount of sorbed substance in %.

At the next stage, it is necessary to determine the dependence of PMC sorption on the concentration of Am:MASGA. For this, the dynamics of sorption of different concentrations of Am:MASGA by microcapsules of the composition (PAH/PSS)3 was determined. The results obtained are shown in Figure 10.

As can be seen from Figure 10, the amount of sorbed substance by capsules (PAH/PSS)_3_ increases linearly with an increase in the Am:MASGA concentration, but the amount of sorbed substance does not change over time and reaches equilibrium in the first 15 min of incubation. In this case, regardless of the concentration of Am:MASGA, about 45% of the initial substance is sorbed. Such an effect may be associated with the electrostatic nature of sorption, since the electrostatic bond is not saturable, and with increasing concentration, the amount of sorbed substance may increase.

**Figure 11:**
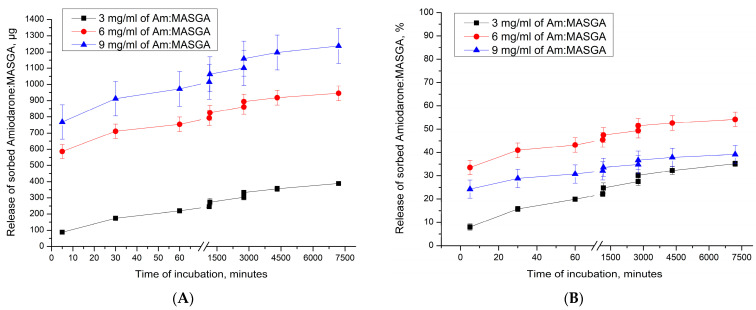
Dynamics of the release of Am:MASGA from microcapsules (PAH/PSS)_3_ incubated at different concentrations of Am:MASGA. (**A**)—the amount of the released substance in μg, (**B**)—the amount of the released substance in%, where 100% was taken as the amount of the included substance.

Subsequently, the release dynamics of Am:MASGA from these microcapsules was studied to determine the release of the substance over time. The results obtained are shown in Figure 11.

As can be seen from Figure 11, there is a gradual release of Am:MASGA from microcapsules (PAH/PSS)_3_. In this case, the amount of the released substance in the first 15 min of incubation depends on the initial amount of the sorbed substance. Thus, PMC adsorbed 1400 μg of the substance released about 100 μg of Am:MASGA during 15 min of incubation, and PMC adsorbed 2500 μg released 600 μg of the substance, etc. There is a gradual release of the substance after 120 h of incubation with the same trend, regardless of the amount of initially sorbed substance, and about 35% ± 5% is released. The result obtained indirectly confirms the hypothesis of the electrostatic nature of the interaction between the polyelectrolyte complex PMC and Am:MASGA.

**Figure 12:**
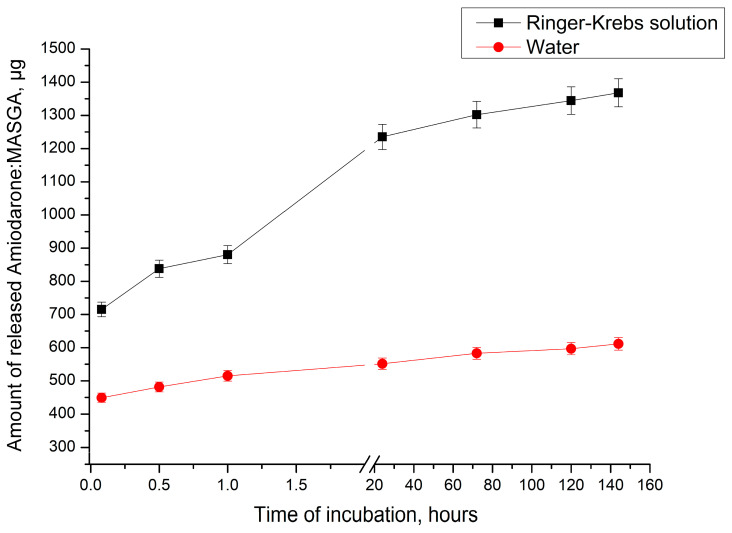
Am:MASHK release dynamics from microcapsules (PAA/PSS)_3_ incubated in water or Krebs-Hanselight solution.

This hypothesis would confirm it is necessary to determine the dynamics of the release of Am:MASGA from microcapsules (PAH/PSS)_3_ during incubation in water and in a solution with an increased ionic strength; for this purpose, the Krebs-Hanselight solution, which is also close to the physiological composition of blood, was used. The results obtained are shown in Figure 12.

As can be seen from Figure 12, the incubation of microcapsules (PAH/PSS)_3_ in Krebs-Hanselight solution releases a significantly greater amount of Am:MASGA than incubation in water. At the same time, the release dynamics in the Krebs-Hanselight solution are approximately 1.8 times higher than in water. The results obtained confirm the hypothesis of the electrostatic nature of the sorption of Am:MASGA by microcapsules (PAH/PSS)_3_, since with an increase in the ionic strength of the solution, the yield of the substance with PMC increases.

## 3. Materials and Methods

### 3.1. Materials

The polyelectrolytes polystyrene sulfonate sodium (PSS) and polyallylamine hydrochloride (PAH) with a molecular mass of 70 kDa, were purchased from Sigma (St. Louis, MO, USA). Na_2_CO_3_, CaCl_2_, MgSO_4_, KH_2_PO_4_, KCl, and NaHCO_3_ were obtained from “Reahim”. Krebs-Hanselight solution, mM concentrations: NaCl—118; KCl—4.7; CaCl_2_—2.5; MgSO_4_—1.2; KH_2_PO_4_—1.1; glucose—5.5; NaHCO_3_—25. MASGA (Sigma-Aldrich, CAS Number 53956-04-0) and Amiodarone purity 95% (Institute of Bioorganic Chemistry named after Acad. A. S. Sadykov, Republic of Uzbekistan, Ts 03535693-35:2020).

### 3.2. Capturing of Amiodarone by CaCO_3_ by Co-Precipitation Method

At a stirring of 0.33 M Na_2_CO_3,_ the 0.33 M CaCl_2_ and Am:MASGA were added. The stirring time was 30 s. The suspension was maintained until complete precipitation of the formed particles [41]. The process of “ripening” of microspherolites was controlled with the help of a light microscope (defined the shape of particles). Following this, the supernatant was decanted, and the precipitate was washed with water. The microparticles were obtained with an average diameter of 4.5 ± 1 μm.

### 3.3. Capturing of Amiodarone by CaCO_3_ by Adsorption Method

This procedure is identical to point 3.2. with the difference that amiodarone was not added at the stage of CaCO_3_ formation. The produced microspherulites were incubated for 1 h in Am:MASGA solution. After that, the supernatant was decanted, and the precipitate was washed with water. The microparticles were obtained with an average diameter of 4.5 ± 1 μm.

### 3.4. Encapsulation of Amiodarone in Polyelectrolyte Microcapsules with Undissolved CaCO_3_ Core (CaCO_3_/PAH/PSS)_n_

The polyelectrolyte microcapsules were obtained by layer-by-layer adsorbing the negatively or positively charged polyelectrolytes onto CaCO_3_ microspherulites, followed by the dissolution of CaCO_3_. At a stirring of 0.33 M Na_2_CO_3_, 0.33 M CaCl_2_ was added. The stirring time was 30 s. Layer-by-layer adsorption of PAH and PSS on the CaCO_3_ microspherulites surface was carried out in polyelectrolytes solutions (concentration 2 mg/mL + 0.5 M NaCl). After each adsorption, the CaCO_3_ particles with adsorbed polyelectrolytes were triple-washed with a 0.5 M NaCl solution, which was necessary to remove unadsorbed polymer molecules. The particles were separated from the supernatant by centrifugation. After applying the required number of layers, the carbonate cores were dissolved in a 0.2 M EDTA solution for 2 h. The resulting capsules were washed three times with water to remove core decay products. The produced polyelectrolyte microcapsules were incubated for 1 h in Am:MASGA solution. After that, the supernatant was decanted, and the precipitate was washed with water. The microcapsules were obtained with an average diameter of 4.5 ± 1 μm.

### 3.5. Encapsulation of Amiodarone in Polyelectrolyte Microcapsules with Dissolved CaCO_3_ Core (PAH/PSS)_n_

This procedure is identical to point 3.4. with the difference that CaCO_3_ core was dissolved in a 0.2 M EDTA solution. The produced polyelectrolyte microcapsules were incubated for 1 h in Am:MASGA solution. After that, the supernatant was decanted, and the precipitate was washed with water. The microcapsules were obtained with an average diameter of 4.5 ± 1 μm.

### 3.6. Determination of Amiodarone Concentration Released from Different Types of Carriers

A definite type of carrier was incubated in a water or Krebs-Hanselight solution for a long period of time before stopping the release of the substance into the solution. At the same time, the concentration of Am:MASGA was detected after each period of time according to a certain experiment by means of precipitation of carrier and sampling of supernatant. After the probe, the supernatant was replaced with distilled water. The concentration of Am:MASGA was measured by the spectrophotometric method (The Cary 60 UV-Vis spectrophotometer, Santa Clara, CA, USA).

### 3.7. Statistical Processing

Each experiment was repeated three times and for each experiment, three trials were taken. Each sample was measured five times and had been calculated as average and standard deviation. The approximation equation for the linear range of detectable phenol concentrations is y = 9 × 10^−5^x + 0.2001, while the approximation reliability coefficient is R^2^ = 0.9888.

## 4. Conclusions

In the course of the work, a comparison was made of different types of encapsulations of amiodarone: monoammonium salt of glycyrrhized acid (molar ratio 1:8) in various types of micron-sized carriers. It has been shown that the method of encapsulating Am:MASGA by the co-precipitation method is not suitable, because no more than 8% of the substance is included in calcium carbonate spherulites.

The possibility of Am:MASGA encapsulation by sorption into microspherulites CaCO_3_ and 2 types of polyelectrolyte microcapsules CaCO_3_/(PAH/PSS)_3_ and (PAH/PSS)_3_ of complex polyelectrolyte organization was also studied. It was found that CaCO_3_ microspherulites and polyelectrolyte microcapsules of CaCO_3_/(PAH/PSS)_3_ composition adsorbed about 30–35% of the initial amount of the substance, while its gradual release is observed with a plateau after 2 h of incubation. Such a short release period of the substance allows us to conclude that these types of capsules is not suitable for the encapsulation of Am:MASGA with its gradual release.

When Am:MASGA is encapsulated by sorption into polyelectrolyte microcapsules of composition (PAH/PSS)_n_ with a complex polyelectrolyte organization, about 50% of the substance from the initial amount is sorbed. In addition, it was shown that an increase in the number of polyelectrolyte layers does not lead to an increase in the sorbed substance, so the optimal composition of PMC of this type is—(PAH/PSS)_3_. It was also shown that during 115 h of incubation, about 25–30% of Am:MASGA is gradually released from microcapsules into the incubation medium, while an increase in the ionic strength of the solution accelerates the process of this release by 1.8 times, which indicates the electrostatic nature of Am:MASGA sorption by polyelectrolyte microcapsules.

Thus, the most suitable type of polyelectrolyte microcapsules for the encapsulation of Am:MASGA with the possibility of its prolonged release are PMCs of composition (PAH/PSS)3 with a complex polyelectrolyte organization.

## Data Availability

Not applicable.
